# Supplementary figures and images for: Overcoming the gender bias in ecology and evolution: is the double-anonymized peer review an effective pathway over time?
Source: PeerJ. 2023 Apr 10;11:e15186. doi: 10.7717/peerj.15186 (PMC10100800; doi:10.7717/peerj.15186)

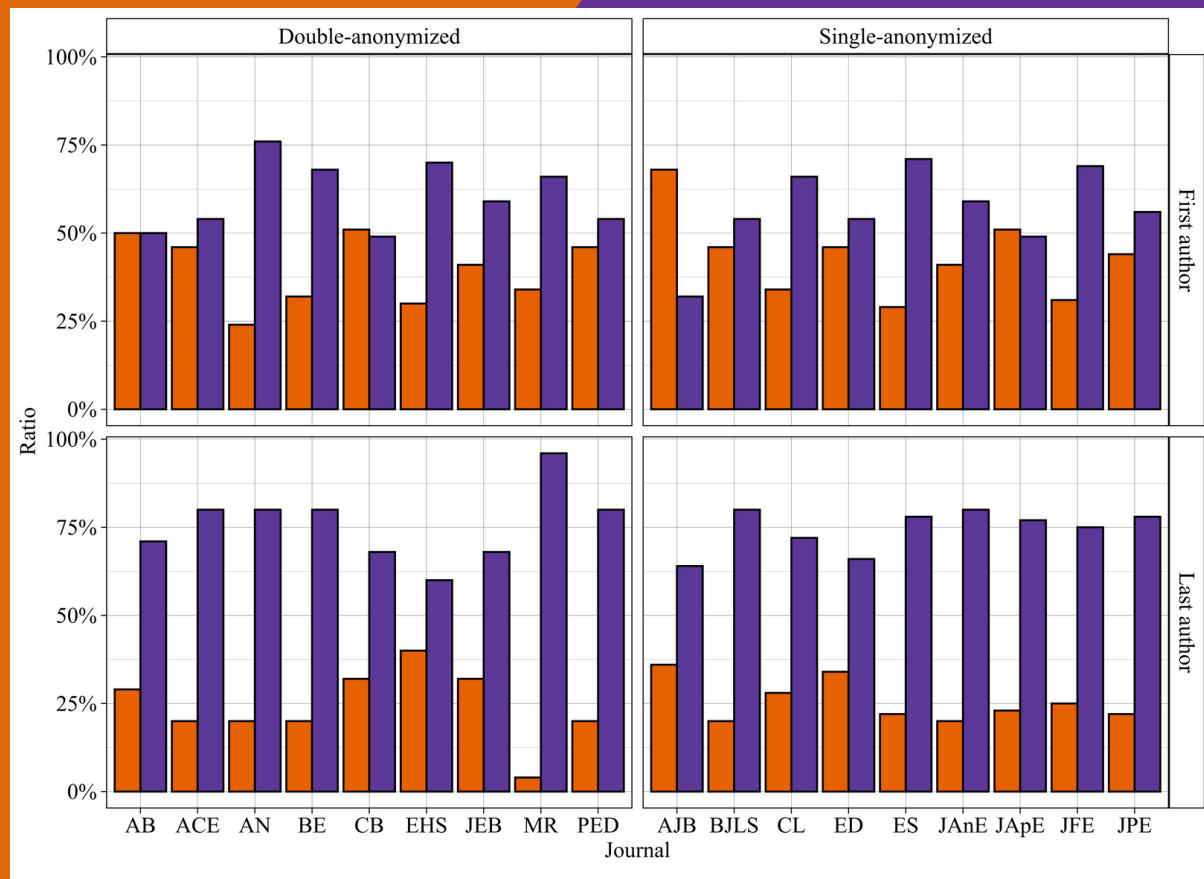

Female Male

Supplement: Supplemental Information 1 [file peerj-11-15186-s001.pdf]
